# Exogenous ethanol induces a metabolic switch that prolongs the survival of *Caenorhabditis elegans* dauer larva and enhances its resistance to desiccation

**DOI:** 10.1111/acel.13214

**Published:** 2020-09-08

**Authors:** Damla Kaptan, Sider Penkov, Xingyu Zhang, Vamshidhar R. Gade, Bharath Kumar Raghuraman, Roberta Galli, Júlio L. Sampaio, Robert Haase, Edmund Koch, Andrej Shevchenko, Vasily Zaburdaev, Teymuras V. Kurzchalia

**Affiliations:** ^1^ Max Planck Institute of Molecular Cell Biology and Genetics Dresden Germany; ^2^ Paul Langerhans Institute Dresden of the Helmholtz Zentrum München at the University Hospital and Faculty of Medicine Carl Gustav Carus of TU Dresden Dresden Germany; ^3^ Institute for Clinical Chemistry and Laboratory Medicine University Clinic and Medical Faculty TU Dresden Dresden Germany; ^4^ Max Planck Institute for the Physics of Complex Systems Dresden Germany; ^5^ Friedrich‐Alexander‐University Erlangen‐Nuremberg Erlangen Germany; ^6^ Max‐Planck‐Zentrum für Physik und Medizin Erlangen Germany; ^7^ Department of Anesthesiology and Intensive Care Medicine, Clinical Sensoring and Monitoring Faculty of Medicine Carl Gustav Carus TU Dresden Dresden Germany; ^8^ Center for Systems Biology Dresden Dresden Germany

**Keywords:** aging, exogenous ethanol, metabolic shift, mitochondrial health, stress resistance

## Abstract

The dauer larva of *Caenorhabditis elegans*, destined to survive long periods of food scarcity and harsh environment, does not feed and has a very limited exchange of matter with the exterior. It was assumed that the survival time is determined by internal energy stores. Here, we show that ethanol can provide a potentially unlimited energy source for dauers by inducing a controlled metabolic shift that allows it to be metabolized into carbohydrates, amino acids, and lipids. Dauer larvae provided with ethanol survive much longer and have greater desiccation tolerance. On the cellular level, ethanol prevents the deterioration of mitochondria caused by energy depletion. By modeling the metabolism of dauers of wild‐type and mutant strains with and without ethanol, we suggest that the mitochondrial health and survival of an organism provided with an unlimited source of carbon depends on the balance between energy production and toxic product(s) of lipid metabolism.

## Graphical abstract

Exogenous ethanol supports the survival of *Caenorhabditis elegans* dauer larvae by inducing a metabolic shift that drives its conversion into sugars, amino acids, and storage lipids, thus promoting mitochondrial integrity and stress resistance. Excessive production of lipids out of ethanol, however, appears to limit the survival due to a buildup of toxic lipids, suggesting that the balance between energy production and lipid metabolism determines the survival in the presence of an unlimited source of carbon.

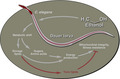

## 1 INTRODUCTION

To survive periods of food deficiency or seasonally induced stress, such as drought or temperature extremes, *Caenorhabditis elegans* enters diapause by forming a specialized larva called a dauer (Riddle, 1988). These non‐feeding larvae remain sealed off from the surrounding environment and so depend on their internal energy sources to support basal physiological processes. Consequently, the amount of stored energy is thought to determine how long a dauer can survive without food and also its ability to resist external stress (Riddle, 1988).

The metabolism and morphology of dauers and reproductive larvae differ significantly. The overall metabolic rate of dauers is much lower, anabolism is kept at minimal levels, and the only source of detectable metabolic activity is very slow catabolism of internal energy stores such as triglycerides (TAGs) (Erkut & Kurzchalia, 2015). In contrast to reproductive stages, dauers display a tightly regulated shift from the TCA cycle and high OXPHOS to glyoxylate shunt, leading to excessive gluconeogenesis (Erkut, Gade, Laxman, & Kurzchalia, 2016; Penkov et al., 2020; Wadsworth & Riddle, 1989). Previously, we have shown that dauer larvae consume about 80% less oxygen than a reproductive L3 larva (Erkut et al., 2016). The major source for the production of sugars (e.g., the disaccharide trehalose) is TAGs that are accumulated during dauer formation. Another characteristic of the dauer larva is its thick and tight cuticle (Albert & Riddle, 1988; Cassada & Russell, 1975). This cuticle is resistant even to a strong detergent (1% SDS) and was found to be impermeable to most substances tested (Cassada & Russell, 1975). Thus, it was assumed that the dauer larva is a closed system with a minimal exchange of matter with the environment (Riddle, 1988). However, some hydrophobic dyes can enter the dauer via small openings for sensory neurons, amphids and phasmids, in the regions of pharynx and tail (Peckol, Troemel, & Bargmann, 2001).

Here, we show that external ethanol can enter the dauer larva. Moreover, the larvae have means to metabolize and use it as an energy and carbon source. The ability to metabolize ethanol prolongs the survival time of wild‐type and mutant dauer larvae significantly and makes them resistant to desiccation. We suggest that this is an adaptation to the situation in nature, where *C. elegans* could use ethanol produced by some bacteria and yeast. The death of dauer larvae is preceded by fragmentation and deterioration of mitochondria, but the addition of ethanol delays this process. To explain the mechanism of ethanol‐mediated lifespan extension in dauers, we propose a model in which a toxic component derived from lipids leads to mitochondrial death even in the presence of a practically unlimited energy source.

## 2 RESULTS

### 2.1 Ethanol activates expression of enzymes required for alcohol metabolism in dauer larvae

**FIGURE 1:**
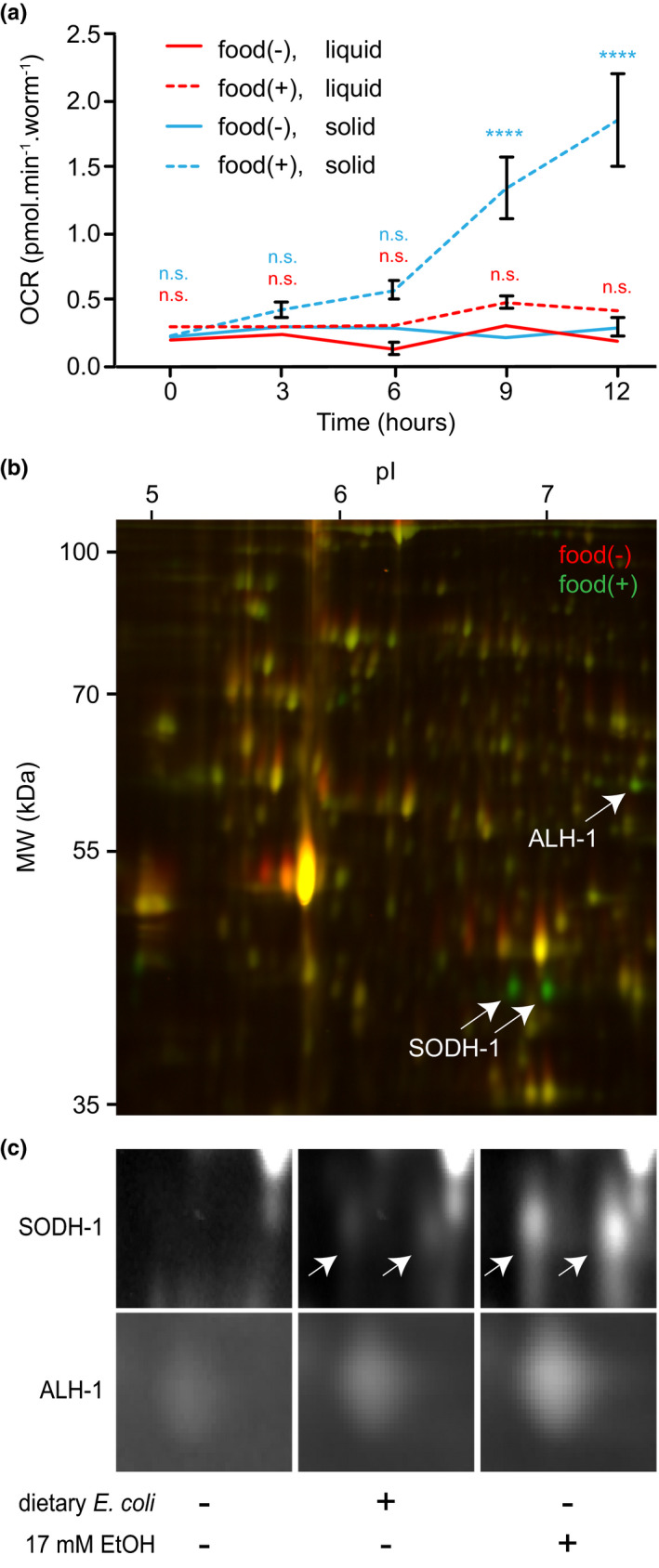
External ethanol induces the expression of alcohol and aldehyde dehydrogenases in dauer larvae. (a) Oxygen consumption rates (OCR) measured in wild‐type dauer larvae upon the addition of food on solid or in liquid growth medium, compared to non‐fed worms. Note that only worms incubated with food on solid medium exit from dauer state and resume growth. Error bars, ±*SD*. ****p < 0.0001; ns—no significant difference determined by two‐way analysis of variance. Color labels of statistical difference signify the contrasts between: red—food(−) liquid versus food(+) liquid; blue—food(−) solid versus food(+) solid. (b) 2D‐Difference gel electrophoresis (2D‐DIGE) of proteins derived from worms treated or untreated with food in liquid medium. Representative images of two experiments. (c) 2D‐DIGE close‐up of SODH‐1 and ALH‐1 in untreated, food (*Escherichia coli*)‐treated and ethanol‐treated wild‐type dauers in liquid medium. Representative images of two experiments.

The effect of ethanol on dauer larvae was noticed while investigating their exit from the arrested state. When dauer larvae are placed on a plate (solid medium) with food, a so‐called “food signal” initiates an exit program and entry into reproductive growth (Golden & Riddle, 1982, 1984; Mylenko et al., 2016). After several hours on food, mitochondria switched from low to high oxygen consumption rates (OCR) and protein synthesis was fully activated (Figure 1a and Figure S1). In contrast, we found that dauer larvae in a liquid medium, an environment with low oxygen concentration, did not exit even in the presence of food and retained a low OCR (Figure 1a). Under these conditions (i.e., in liquid), however, we observed a strong upregulation of several proteins, as shown by 2D difference gel electrophoresis (2D‐DIGE, Figure 1b). The two most prominent of them were identified as sorbitol dehydrogenase‐1 (SODH‐1) and aldehyde dehydrogenase‐1 (ALH‐1) (Figure 1b). These are members of the alcohol and aldehyde dehydrogenase families, respectively. The same enzymes, involved in ethanol metabolism, have been found to be induced in arrested L1 larva upon ethanol treatment (Artyukhin, Yim, Cheong Cheong, & Avery, 2015; Patananan, Budenholzer, Eskin, Torres, & Clarke, 2015). Since the food contains 0.1% (17 mM) ethanol, due to the requirement to dissolve cholesterol in ethanol during food preparation, we hypothesized that the presence of ethanol in the medium induced the changes in protein expression. Figure 1c shows that indeed the presence of ethanol induced SODH‐1 and ALH‐1 production much more strongly than dietary *Escherichia coli*. The weak ability of bacteria *per se* to induce SODH‐1 and ALH‐1 could be used by worms to prepare for increased alcohol production by microorganisms or a response to bacterial metabolites. We conclude that the strong SODH‐1 and ALH‐1 upregulation is not part of the exit program but a consequence of the addition of ethanol to the medium.

### 2.2 Ethanol induces a metabolic switch that enhances its utilization as a carbon source

**FIGURE 2:**
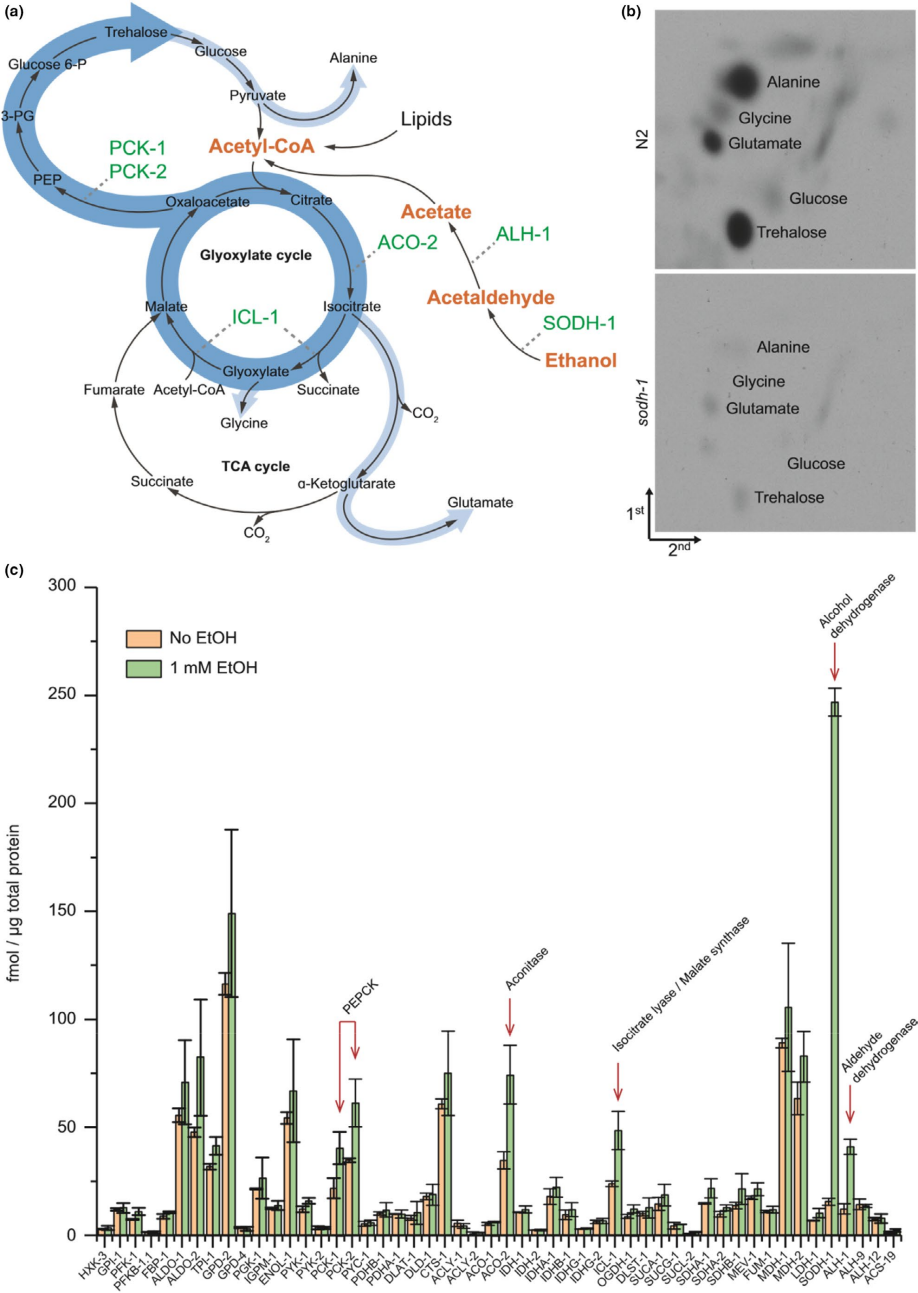
Externally added ethanol induces a metabolic switch that promotes its conversion into carbohydrates and amino acids. (a) General scheme of ethanol metabolism in dauer larvae based on analysis of carbon incorporation (see panel b). (b) 2D‐TLC of aqueous extracts containing hydrophilic metabolites from ^14^C‐ethanol‐labeled wild‐type dauers. Representative images of two experiments. (c) Absolute quantification via MS Western of enzymes involved in ethanol metabolism, TCA cycle, glyoxylate shunt, glycolysis, gluconeogenesis, pyruvate, and acetyl‐CoA metabolism in dauers treated or untreated with ethanol. Data obtained from two biological and two technical replicates. Error bars, ±*SD*. The positions of six enzymes that are upregulated upon ethanol treatment are depicted in panel a (in green).

Since SODH‐1 and ALH‐1 can transform ethanol to acetaldehyde and subsequently to acetate, we hypothesized that ethanol could penetrate the cuticle of the dauer larva and induce a switch that allows it to be used as an energy source. It is known that dauers operate in gluconeogenic mode and produce sugars/carbohydrates, mainly trehalose, from lipid stores (Figure 2a) (Erkut et al., 2016). Thus, the major metabolic pathway is the glyoxylate shunt, which utilizes two acetate molecules to produce malate and succinate. Malate can be oxidized to oxaloacetate and enter the gluconeogenic pathway. Thus, ethanol via acetate could be incorporated into trehalose and used further in glycolysis to produce ATP (Erkut et al., 2016). Indeed, 2D‐TLC of metabolites derived from dauer larvae incubated with ^14^C‐labeled ethanol showed incorporation of the label into several compounds (Figure 2b). In our previous study, we have identified all these compounds based on the chromatographic behavior of synthetic standards (Erkut et al., 2016). The strongest incorporation was observed in trehalose (Figure 2b). The amino acids alanine, glycine, and glutamate were also labeled (Figure 2b). All these compounds, derived mainly via gluconeogenesis, could be used as an energy source. The labeling was clearly SODH‐1 dependent, as a mutant strain bearing a deletion in *sodh*‐*1* incorporated only negligible amounts of radioactivity (Figure 2b).

The influx of ethanol‐derived carbon into the production of trehalose and amino acids might result in a change of the steady‐state levels of some of these compounds over time. As evidenced by HPLC‐MS analysis, the levels of trehalose substantially increased upon ethanol treatment of wild‐type dauers (Figure S2A). In contrast, HPLC‐MS of amino acids that could be derived from ethanol (e.g., alanine, serine, glutamate, glutamine) showed no increase upon ethanol treatment (Figure S2B–E). This can be explained by a rise in the turnover of these amino acids. Indeed, the levels of urate, a product of breakdown of purines, showed elevated levels in ethanol‐treated dauers (Figure S2F). This signified a higher turnover of amino acids used for purine syntheses, such as serine, glutamine, and glycine.

According to our results, an upregulation of SODH‐1 and ALH‐1 could facilitate the utilization of ethanol as a source of carbon for the synthesis of sugars and amino acids. To determine whether ethanol also induces changes in the levels of other enzymes that act downstream of acetate in the metabolic pathway, we used the LC‐MS/MS method of MS Western (Kumar et al., 2018), a method for simultaneous quantification of the absolute (molar) amount of multiple proteins. Using this method, we have recently shown that a shift in the molar abundances of several metabolic enzymes drives the transition from the TCA cycle to gluconeogenesis that worms undergo during dauer formation (Penkov et al., 2020). Hence, we quantified the molar amount of 48 enzymes involved in the TCA cycle, glyoxylate shunt, glycolysis, gluconeogenesis, pyruvate, and acetyl‐CoA metabolism in dauers treated with 1 mM ethanol compared to untreated animals. We also included SODH‐1 and ALH‐1, as well as two other aldehyde dehydrogenases that were also present in high amounts in dauer proteomes (ALH‐9 and ALH‐12, Figure S3). Overall, most of the proteins remained unchanged (Figure 2c). However, there was a pronounced upregulation of some enzymes acting in key reactions for the conversion of ethanol to carbohydrates (Figure 2a,c). SODH‐1 showed the highest increase (14.5‐fold), making it the most abundant among the tested proteins in ethanol‐treated worms (Figure 2a,c). The second highest increase was displayed by ALH‐1 (3.2‐fold, Figure 2a,c). The abundance of the other aldehyde dehydrogenases did not change (Figure 2c and S3). Combined with the visible upregulation of a single band corresponding to an aldehyde dehydrogenase according to 2D‐DIGE (Figure 1b), we concluded that ALH‐1 was the only member of the aldehyde dehydrogenase family specifically induced by ethanol in dauers. Downstream of acetate production, the changes were more moderate. However, they reflected a clear tendency for enhanced glyoxylate pathway and gluconeogenesis. The consecutive conversion of acetyl‐CoA‐derived citrate into isocitrate, glyoxylate, and malate was stimulated by increases in the levels of aconitase (ACO‐2, 2.6‐fold) and the glyoxylate pathway enzyme ICL‐1 (2.5‐fold, Figure 2a,c). Furthermore, gluconeogenesis was supported by an increase in the two phosphoenolpyruvate carboxykinases (PEPCKs) PCK‐1 and PCK‐2 (2.2‐fold each) and the aldolase ALDO‐2 (2‐fold, Figure 2a,c). Thus, a metabolic switch involving several key enzymes enhances the metabolism of ethanol through glyoxylate shunt and gluconeogenesis, providing carbon atoms for the synthesis of sugars and amino acids, as well as for energy production.

### 2.3 Ethanol replenishes lipid storage, prolongs survival, and enhances stress resistance

**FIGURE 3:**
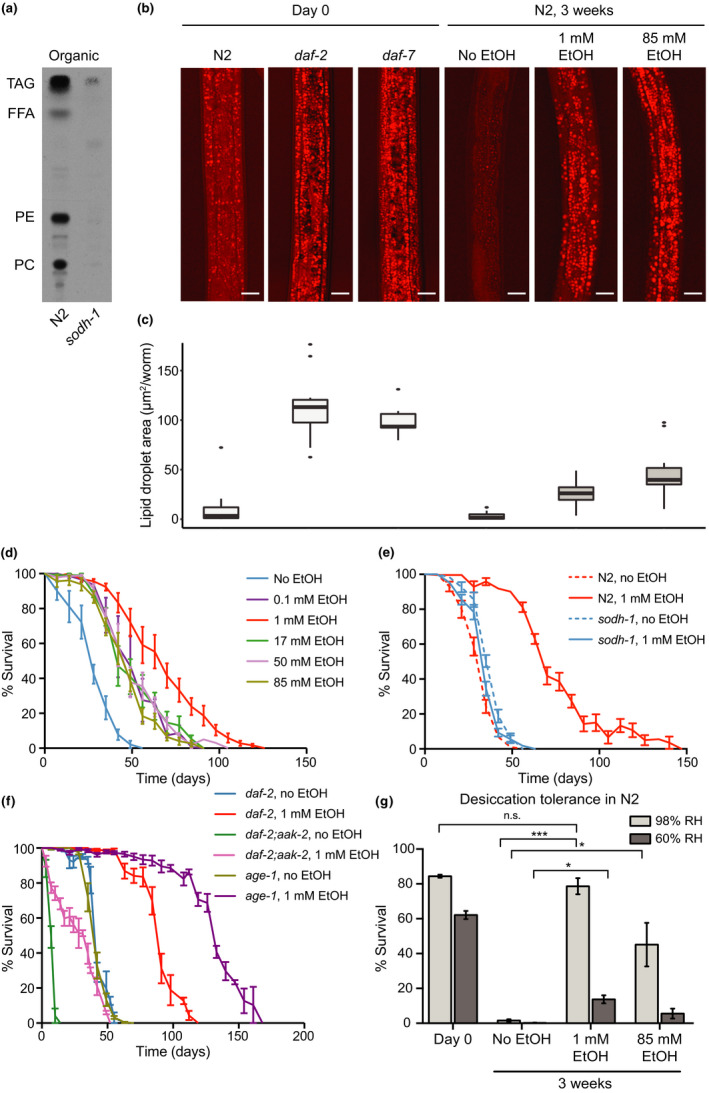
Ethanol replenishes the lipid storage and increases the survival and stress resistance of the dauer larvae. (a) 1D‐TLC of organic extracts containing lipids from ^14^C‐ethanol‐labeled wild‐type dauers. TAG—triacylglycerols, FFA—free fatty acids, PE—phosphatidylethanolamines, PC—phosphatidylcholines. Representative images of two experiments. (b) CARS microscopy revealing the accumulation of lipid droplets in ethanol‐treated and untreated dauers at different time points. Scale bars, 10 μm. (c) Total area of the lipid droplets visualized by CARS in ethanol‐treated and untreated dauers at different time points as seen in panel b. Means of at least two experiments with 9‐21 animals. N2 (day 0)–*daf*‐*2* (day 0): *p* < 0.0001; N2 (day 0)–*daf*‐*7* (day 0): *p* < 0.0001; N2 (day 0)–N2 (week 3, no EtOH): not significant; N2 (day 0)–N2 (week 3, 1 mM EtOH): not significant; N2 (day 0)–N2 (week 3, 85 mM EtOH): *p* < 0.001; N2 (week 3, no EtOH)–N2 (week 3, 1 mM EtOH): *p* < 0.01; N2 (week 3, no EtOH)–N2 (week 3, 85 mM EtOH): *p* < 0.0001 according to one‐way ANOVA with Tukey HSD. (d) Survival rates of wild‐type dauers (N2) untreated or incubated with different concentrations of ethanol. Error bars, ±*SD* of at least two experiments with at least three biological replicates. One‐way analysis of variance of untreated dauers (no EtOH) compared to worms treated with following concentrations of ethanol showed: 0.1 mM: *p* = 0.0009; 1 mM: *p* < 0.0001; 17 mM: *p* = 0.0001; 50 mM: *p* < 0.0001; 85 mM: *p* < 0.0001. (e) Survival rates of wild‐type and *sodh*‐*1*‐deficient dauers untreated or treated with ethanol. Error bars, ±*SD* of at least two experiments with at least three biological replicates. Two‐way analysis of variance determined following difference between the groups: N2 no EtOH versus N2 1 mM EtOH: *p* < 0.0001; *sodh*‐*1* no EtOH versus *sodh1* 1 mM EtOH: *p* = 0.0016; *sodh*‐*1* no EtOH versus N2 no EtOH: *p* = 0.0004; *sodh*‐*1* 1 mM EtOH versus N2 1 mM EtOH: *p* < 0.0001. (f) Survival rates of *daf*‐*2*, *daf*‐*2*;*aak*‐*2*, and *age*‐*1* mutant dauers untreated or treated with ethanol. Error bars, ±*SD* of at least two experiments with at least three biological replicates. Two‐way analysis of variance determined following difference between the groups: *daf*‐*2* no EtOH versus *daf*‐*2* 1 mM EtOH: *p* < 0.0001; *daf*‐*2*;*aak*‐*2* no EtOH versus *daf*‐*2*;*aak*‐*2* 1 mM EtOH: *p* < 0.0001; *daf*‐*2* no EtOH versus *daf*‐*2*;*aak*‐*2* no EtOH: *p* < 0.0001; *daf*‐*2* 1 mM EtOH versus *daf*‐*2*;*aak*‐*2* 1 mM EtOH: *p* < 0.0001; *age*‐*1* no EtOH versus *age*‐*1* 1 mM EtOH: *p* < 0.0001; *age*‐*1* no EtOH versus *daf*‐*2* no EtOH: *p* = 0.5544; *age*‐*1* 1 mM EtOH versus *daf*‐*2* 1 mM EtOH: *p* < 0.0001. (g) Desiccation tolerance of wild‐type dauers untreated or incubated with ethanol followed by desiccation at a different relative humidity (RH). Error bars, ±*SD*. *** *p* < 0.001; * *p* < 0.1; ns—no significant difference determined by one‐way analysis of variance.

The production of sugars and amino acids from ethanol boosts the renewal of metabolites and maintains the overall metabolic network active. However, the excess carbon from ethanol provided *ad libitum* might also be stored as an energy reserve. It has long been presumed that, in the dauer state, worms degrade but do not synthesize lipids in bulk amounts due to an overall inhibition of the anabolism (Riddle, 1988). The observation that dauer larvae can metabolize external ethanol raised a fundamental question: Whether dauer larvae are capable of anabolic reactions and whether they can build up energy stores (e.g., storage lipids) if provided with excess amounts of an external carbon source. To address this question, we tested whether the carbon from ^14^C‐labeled ethanol could be traced to lipids. Astoundingly, we observed high incorporation, also SODH‐1 dependent, of the radioactivity into lipids (Figure 3a). A TLC of the lipid extract displayed a regular pattern of newly synthesized lipids derived from ethanol. Among them, TAGs were the most abundant (Figure 3a). We, therefore, asked whether incubation with ethanol indeed leads to a bulk accumulation of TAGs in dauers. For this, we applied CARS microscopy of lipid droplets (LDs), the major sites of TAG deposition. Newly generated wild‐type dauers formed under overcrowding/starvation (Day 0) displayed a certain amount of LDs (Figure 3b,c). After 3 weeks without exogenous ethanol, wild‐type dauers showed almost no LDs, whereas incubation with 1 mM or 85 mM ethanol led to a significant increase in LDs (Figure 3b,c). Of note, in order to maintain the ethanol concentration relatively constant, we regularly renewed the medium (see Experimental procedures). Hence, ethanol (via sugars and amino acids) can be used not only as a direct energy source in catabolic reactions, but also as a starting point for anabolic reactions, producing lipid deposits.

Next, we asked whether external ethanol, by providing additional energy source, supports two major purposes of dauer larvae: increased survival time in the absence of food and resistance to environmental stress (e.g., desiccation). Firstly, we investigated the influence of ethanol on the survival of dauer larvae in liquid. Fresh ethanol was added regularly. Normally, in liquid, the life span of a wild‐type dauer was about 4 weeks (Figure 3d). When incubated in 0.1% ethanol (i.e., 17 mM, the concentration used initially in food), the life span increased to about 6 weeks. The most optimal ethanol concentration was found to be 1 mM (Figure 3d), which doubled the life span. The higher concentrations of ethanol (50 or 85 mM) did not increase the effect and were slightly toxic (Figure 3d). In agreement with the inability of *sodh*‐*1* mutants to metabolize ethanol, we observed no increase in the survival in these mutants upon treatment with ethanol (Figure 3e). Thus, the life‐extending effect of ethanol appears to be caused by products of its metabolism and not by ethanol itself (e.g., by quenching of ROS).

So far, the survival of wild‐type dauers formed in response to overcrowding/starvation has been presented. Next, we analyzed dauer larvae of Daf‐c mutants, which carry conditional, temperature‐activated mutations that induce dauer formation even on ample food and at low population density (Fielenbach & Antebi, 2008). Because they can form dauers under unrestricted food supply, they might be able to store more lipids during active feeding preceding the dauer arrest (Riddle, 1988). Indeed, dauers of Daf‐c mutants of *daf*‐*2* and *daf*‐*7* (of the insulin and the TGF‐β signaling pathways, respectively) contained larger amounts of LDs from the beginning (Figure 3b,c). Consistently, *daf*‐*2* mutants survived longer than the wild‐type worms (Compare Figure 3d,f; Figure S4). However, the increase in the survival rate in *daf*‐*2* was rather moderate, suggesting that the ability to secure a source of energy such as ethanol could be crucial for dauer survival in the wild.

Next, we asked whether the effect of ethanol on the life span of dauers depends on signaling factors that regulate the energy stores and the overall energy expenditure in the dauer state. First, we tested whether ethanol can prolong the survival of short‐lived dauers of *daf*‐*2*;*aak*‐*2* strain lacking functional AAK‐2/AMPKα, a major regulator of cell metabolism. The dauers of this strain deplete their energy stores very fast, enter a starvation‐like state, and die prematurely (Narbonne & Roy, 2009; Penkov et al., 2020). However, on ethanol, these mutants lived almost 4 weeks instead of 7‐8 days (Figure 3f). Note that the survival rate of *daf*‐*2*;*aak*‐*2* on ethanol was still much lower compared to *daf*‐*2* (Figures 3f, S4) or wild‐type dauers (Figures 3d, S4). This suggested that AMPK mutant dauers may be more sensitive to alcohol toxicity. Next, we tested dauers with mutations in *age*‐*1*, which encodes a conserved class I PI3‐kinase participating in the insulin pathway (Fielenbach & Antebi, 2008). *age*‐*1* is an important regulator of fat accumulation in the dauer state (Ogg & Ruvkun, 1998). Without the provision of ethanol, these dauers did not live longer than *daf*‐*2* dauers (Figure 3f). In contrast, ethanol tripled their life span, making them survive far longer than *daf*‐*2* or wild‐type dauers on ethanol (Figure 3f, S4). The fact that *age*‐*1* dauers display higher survival rates than *daf*‐*2* only when ethanol is added suggests that PI3‐kinases optimize ethanol metabolism. Together, our results suggest that AMPK and PI3‐kinase signaling modulates the utilization of external energy sources in dauer larvae.

In addition to the extension of survival time, we wondered if ethanol could also increase the stress resistance of dauer larvae. Previously, we have shown that in preparation for entry into a dry state (anhydrobiosis), dauers use storage lipids as a source of carbon to produce trehalose, which is required for survival during desiccation (Erkut et al., 2011, 2016). As shown in Figure 3g, the ability of dauer larvae to survive even mild desiccation (98% relative humidity) after 3 weeks, when the lipid stores were exhausted (Figure 3b), was negligible. In contrast, larvae exposed to ethanol deposited large amounts of LDs and had fully restored survival under the same conditions (Figure 3g). Moreover, a considerable fraction of the ethanol‐treated animals survived even harsh desiccation (60% relative humidity) (Figure 3g). These data suggest that ethanol augments the desiccation tolerance of dauers by replenishing the lipid stores required for trehalose production. Hence, we hypothesized that AMPK mutant dauers must become sensitive to desiccation much earlier than larvae with intact AMPK due to the rapid loss of lipid reserves. Indeed, as early as the third day of arrest, almost no animals of the *daf*‐*2*;*aak*‐*2* strain recovered from desiccation (Figure S5). In contrast, some of the *daf*‐*2*;*aak*‐*2* worms treated with 1 mM ethanol did survive, while the treatment with 85 mM ethanol fully restored their initial survival rate (Figure S5). Thus, external ethanol can deliver energy and substrates essential for the survival and stress resistance of the non‐feeding dauer.

### 2.4 Ethanol slows the deterioration of mitochondria

**FIGURE 4:**
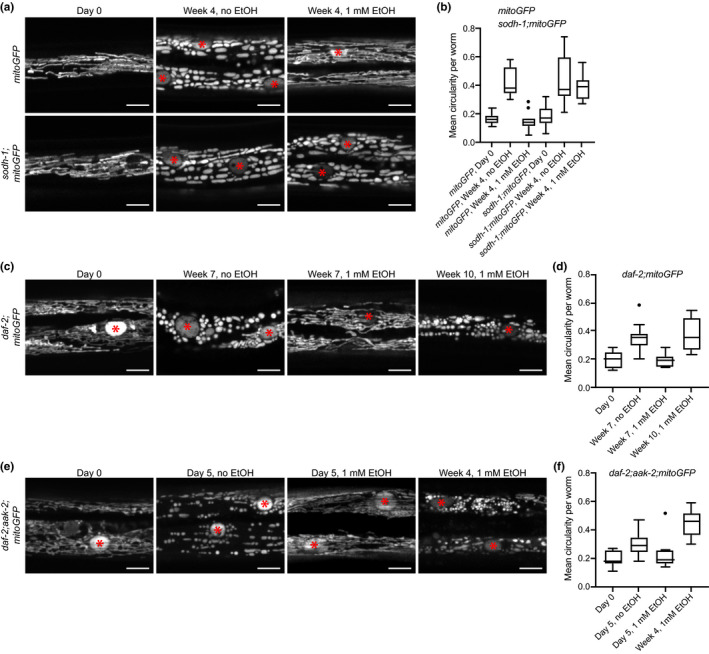
Ethanol delays the deterioration of mitochondria that precedes the death of the dauer larva. (a) Confocal micrographs of eGFP‐marked body wall muscle mitochondria in *mitoGFP* and *sodh*‐*1*;*mitoGFP* strains treated or untreated with ethanol. Representative images of two experiments and at least 7 animals per experiment. Red asterisks, nuclei. Scale bars, 5 μm. (b) Mean circularity of mitochondria in *mitoGFP* and *sodh*‐*1*;*mitoGFP*. Nuclei were excluded before the analysis. *mitoGFP*, Day 0 versus *mitoGFP*, Week 4, no EtOH: *p* < 0.0001; *mitoGFP*, Day 0 versus *mitoGFP*, Week 4, 1 mM EtOH: not significant; *mitoGFP*, Day 0 versus *sodh*‐*1*;*mitoGFP*, Day 0: not significant; *sodh*‐*1*;*mitoGFP*, Day 0 versus *sodh*‐*1*;*mitoGFP*, Week 4, no EtOH: *p* < 0.0001; *sodh*‐*1*;*mitoGFP*, Day 0 versus *sodh1*;* mitoGFP*, Week 4, 1 mM EtOH: *p* < 0.0001 according to one‐way ANOVA with Tukey HSD. (c) Muscle mitochondria in *daf*‐*2*;*mitoGFP* treated or untreated with ethanol. Representative images of two experiments and at least 7 animals per experiment. Red asterisks, nuclei. Scale bars, 5 μm. (d) Mean circularity of mitochondria in *daf*‐*2*;*mitoGFP*. Nuclei were excluded before the analysis. Day 0 versus Week 7, no EtOH: *p* < 0.0001; Day 0 versus Week 7, 1 mM EtOH: not significant; Day 0 versus Week 10, 1 mM EtOH: *p* < 0.0001 according to one‐way ANOVA with Tukey HSD. (e) Muscle mitochondria in *daf*‐*2*;*aak*‐*2*;*mitoGFP* treated or untreated with ethanol. Representative images of two experiments and at least 7 animals per experiment. Red asterisks, nuclei. Scale bars, 5 μm. (e) Mean circularity of mitochondria in *daf*‐*2*;*aak*‐*2*;*mitoGFP*. Nuclei were excluded before the analysis. Day 0 versus Day 5, no EtOH: *p* = 0.0051; Day 0 versus Day 5, 1 mM EtOH: not significant; Day 0 versus Week 4, 1 mM EtOH: *p* < 0.0001 according to one‐way ANOVA with Tukey HSD.

The prolonged survival of dauers supplied with ethanol could be explained with increased fitness of cells due to a replenishment of the energy reserves. Therefore, we decided to investigate the cellular basis of the effect of ethanol. Without an external energy source, the survival time of a dauer should be determined by the amount of internal energy. The exhaustion of these deposits ends with cell and organismal death often linked to the status of mitochondria (Galluzzi, Kepp, & Kroemer, 2012; Kroemer, Dallaporta, & Resche‐Rigon, 1998). We, therefore, analyzed a strain expressing a fluorescent reporter in muscle mitochondria (mitoGFP), which allowed us to monitor mitochondrial morphology. In young wild‐type dauers (Day 0), mitochondria had an elaborate tubular structure (Figure 4a), typical of their normal operational state (Westermann, 2012). After 4 weeks without ethanol, before dauers died, mitochondria appeared deteriorated and tubules were fragmented into non‐uniform spheres and ellipsoids (Figure 4a), as evidenced by the increased circularity of the mitoGFP‐positive objects (Figure 4b). Incubation with ethanol delayed this process, and after 4 weeks, mitochondria were almost indistinguishable from those observed at Day 0 (Figure 4a,b). This delay depended on ethanol metabolism since *sodh*‐*1* mutants supplemented with ethanol displayed similar mitochondrial deterioration to that observed in the absence of ethanol (Figure 4a,b).

As mentioned above, the ethanol source was renewed regularly. Hence, under the experimental conditions, exogenous ethanol is a seemingly unlimited source of energy. Nevertheless, dauers die. Thus, the absence of energy may not be the only reason for the deterioration of mitochondria. We analyzed the status of mitochondria in *daf*‐*2* and *daf*‐*2*;*aak*‐*2* mutants carrying mitoGFP after treatment with ethanol. In both strains, ethanol delayed the deterioration of mitochondria significantly (Figure 4c–f). However, after 10 weeks in ethanol, before they died, *daf*‐*2* dauers displayed fragmented mitochondria despite the presence of ethanol (Figure 4c,d). The mitochondria of *daf*‐*2*;*aak*‐*2* dauers were similarly fragmented before their death despite the presence of ethanol (Figure 4e,f). This suggests that at a later stage, a second, energy‐independent factor may be responsible for mitochondrial dysfunction and lethality in long‐ and short‐lived dauers.

### 2.5 The effect of ethanol on survival can be mathematically predicted based on the balance between energy production and toxicity

We then asked what could be the factor that limits the survival of dauers when an unlimited source of energy and carbon is provided. One possible factor is the depletion of another element than carbon. The medium used in our assays is rich in phosphates and sulfates and contains a mix of trace metals (see Experimental procedures). Thus, we set out to test whether a source of nitrogen would complement the treatment with ethanol. To this end, we tested a mixture of proteinogenic amino acids and ammonium chloride, as well as vitamins (see Experimental procedures). Neither the individual mixtures nor the combination of both augmented the effect of ethanol (Figure S6). Hence, provided that these compounds are taken up by dauer larvae, none of them seems to limit the survival within the experimental time frame. This notion makes the stepwise resource scarcity less probable as an explanation of our observations.

We, therefore, hypothesized that the metabolism of ethanol could also have negative effects on the survival that gradually cancel out the benefits. To address this hypothesis, we built a minimal mathematical model of the *C. elegans* dauer metabolic pathway and applied it to all our experimental observations in wild‐type and mutant strains, with and without external ethanol.

**FIGURE 5:**
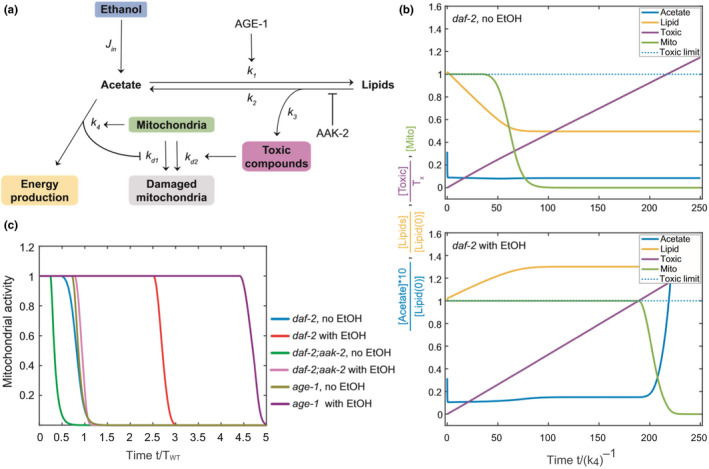
Mathematical modeling of ethanol utilization and survival rates in dauer larvae. (a) A simplified metabolic network that serves as a base for the mathematical model of ethanol utilization and survival rates. Ethanol is a source of acetate, further used for energy production or lipid storage. Lipid catabolism contributes to energy production via synthesis of acetate, but due to metabolic leakage, it can lead to the emergence of toxic compounds. The mitochondrial activity is used as an approximation of survival rate. Its relationship with energy production is bidirectional: mitochondrial activity requires and is required for conversion of acetate into energy. On the other hand, mitochondrial activity is negatively affected by the toxic products of lipid catabolism. The model uses all reaction rates displayed in the scheme plus the presence of ethanol and lipid deposits. It tests if there is a region in the parameter space where, by having all parameters fixed and only changing those that would correspond to a certain mutation or a presence or absence of ethanol and lipids, the changes in the life span will be consistent with the experimental ones. (b) Model‐predicted survival rates co‐plotted with several other trends (see below) as exemplified for the *daf*‐*2* strain untreated or treated with ethanol. “Acetate” is the combined entity representing the free acetic acid and the acetyl‐CoA produced in the pathway. “Lipid” represents the bulk complex lipids, mainly TAGs, derived from the acetate component. “Toxic” comprises the putative lipid‐derived toxic compound(s). “Mito” is defined by the degree of activity of the mitochondria and serves as a proxy to the survival rate. Note that after the mitochondrial activity (“Mito”) falls to zero, the model still exhibits some dynamics, but this is out of the biologically relevant regime. (c) Model‐predicted survival rates for *daf*‐*2*, *daf*‐*2*;*aak*‐*2*, and *age*‐*1* untreated or treated with ethanol. Compare with experimental survival rates of the same strains in Figure 3f

As the basis for the mathematical model, we took a streamlined version of the metabolic pathway shown in Figure 2a. External ethanol can be metabolized to “Acetate”, which is used in “Energy production” and also to replenish the storage of “Lipids” (Figure 5a). These two reaction channels are linked to two possible mechanisms that affect the lifespan of the dauer. Experimental data suggest that the state of mitochondria may serve as a valid proxy for the well‐being of the worm. Here, we assume that mitochondria start to get damaged if the flux of “Acetate” to “Energy production” drops below a certain minimum threshold. That would be the primary cause of death in dauers when no external ethanol is provided and the lipid storage gets depleted. With external ethanol, we hypothesize that an additional damage mechanism is linked to the production of toxic compounds during catabolism of lipids (“Lipid” to “Acetate” transition). While having an infinite energy source, the major cause of death becomes the accumulation of these toxic compounds above a certain threshold. The model could be formalized as a system of differential equations describing the chemical reaction network and solved numerically (Supporting information), and it replicated the behavior of dauers with and without ethanol (Figure 5b,c).

Next, we tested the predicted behavior of *daf*‐*2*;*aak*‐*2* dauers, where lipid catabolism was aberrantly high (Narbonne & Roy, 2009; Penkov et al., 2020). The model confirms that a higher rate of lipolysis leads to premature mitochondrial deterioration because the energy reserves are rapidly depleted (Figure 5a,c, and Figure S7A,B). However, ethanol addition only partially rescues the mitochondria, because the fast lipolysis makes the rate of toxic buildup higher. That way, although the dauers may have enough energy, they pass the damage threshold early and die prematurely (Figure 5c and Figure S7A,B).

We also asked whether the model could explain the greatly extended survival of *age*‐*1* dauers on ethanol in comparison with wild‐type and *daf*‐*2* dauers. In the model, this could be possible when the rate of toxic buildup is lower. Such an effect is achieved if the lipogenesis rate is low and acetate is mostly used in carbohydrate production without rapid elevation of toxic products (Figure 5a,c, and Figure S7C,D). In this regime, worms will be predicted to have lower lipid storage. To experimentally test this, we measured the incorporation of^ 14^C‐ethanol into lipids in *age*‐*1*. Indeed, consistent with the model, *age*‐*1* dauers accumulated significantly lower amounts of labeled lipids compared to wild‐type animals (Figure S8).

Figure 5c summarizes the results of the model showing the lifespans of all mutants that we tested experimentally with and without external ethanol. The remarkable agreement with experimental data (Figure 3f) that we could achieve with this rather simple model suggests that we could identify the most relevant conceptual mechanisms of dauer survival.

## 3 DISCUSSION

Data presented in our study show that exposure of dauers to ethanol can support extended survival and resistance to stress. This ability has a very important physiological implication. *C. elegans* is often found in the proximity of rotting fruits and plant stems (Felix & Duveau, 2012). Thus, in its natural surroundings, dauers could exploit ethanol produced locally by many yeast or bacterial strains and increase their energy deposits. Notably, the optimal concentration for the prolongation of survival or desiccation tolerance is quite low (1 mM; ca. 0.005%), allowing dauers to capture naturally occurring ethanol from sources of low abundance. Note that, in nature, ethanol can reach two orders of magnitude higher concentrations in some fruits (Dudley, 2004). Thus, in times of food scarcity or overcrowding, as well as during preparation for the seasonal (e.g., winter) diapause, dauers may opportunistically use ethanol as a source of energy.

Our results show that the utilization of external ethanol involves a metabolic switch initiated by a massive increase in alcohol‐metabolizing enzymes. The first enzyme in the pathway, SODH‐1, represented similarly to the average of all other enzymes (0.9 fold) at basal dauer state, becomes the most abundant enzyme at 7.8‐fold higher levels than the average upon ethanol treatment. This effect channels ethanol into the production of acetyl‐CoA. Downstream of acetyl‐CoA, the metabolic switch consists of two separate modules: (a) intensification of the glyoxylate shunt and gluconeogenesis for production of sugars and amino acids and (b) a shift to lipogenesis. The combined effect of the two modules (gluconeogenesis and lipogenesis) allows dauers not only to produce energy and to boost the metabolic network *ex tempore*, but also to store the excess carbon derived from ethanol into lipid reserves. Of note, in the first module, the incorporation of acetate‐derived carbon into amino acids must be accompanied by transaminations that consume other amino acids. Hence, the metabolic switch must be coordinated with the amino acid homeostasis to be beneficial.

The two modules use the same substrate (acetyl‐CoA), making them effectively two competing branches of the pathway. These two branches seem to be differentially regulated, allowing for control of the balance between energy production and storage. This notion is supported by the fact that *age*‐*1* mutants have diminished incorporation of ethanol into lipids but not into hydrophilic metabolites, suggesting that the balance between energy production and storage is under the control of PI3K signaling. One interesting aspect of our study is that a mutation in *age*‐*1* is more beneficial for the survival upon ethanol treatment than a mutation in *daf*‐*2*, encoding an insulin/IGF‐1 receptor homolog. Given that *daf*‐*2* acts directly upstream of *age*‐*1* (Murphy & Hu, 2013), we should expect similar outcomes of the two mutations if they operate in a linear pathway. However, as class I PI3‐kinases act in response to many other receptor tyrosine kinases and G protein‐coupled receptors (Bilanges, Posor, & Vanhaesebroeck, 2019), AGE‐1 may integrate stimuli from various receptors in response to exogenous ethanol. The dissection of the signaling cascade is, thus, an important aspect of future investigations.

It is important to note that this metabolic switch must be balanced with the signaling pathways that govern larval growth and development. In previous studies, we have shown that deregulation of the dauer metabolic mode toward pathways that support anabolism (e.g., production of more NADPH, enhancing the TCA cycle) could lead to a transition from quiescence to growth (Penkov et al., 2015, 2020). However, ethanol‐treated dauer larvae remain in a growth‐arrested state, which points to a fine‐tuning regulatory mechanism that decouples anabolism from growth. One possible explanation is that the increase in glyoxylate shunt diminishes the isocitrate levels that can be used for NADPH production by the isocitrate dehydrogenase IDH‐1. It is known that NADPH generated by IDH‐1 is used as a co‐factor for the synthesis of steroid hormones, dafachronic acids, that inhibit dauer state (Penkov et al., 2015). Thus, higher glyoxylate shunt activity may prevent exit from the dauer state by lowering the production of dafachronic acids. Although the components of this mechanism remain to be elucidated, they may be encoded into neuroendocrine circuits that sense nutrition, population density, oxygen levels, etc.

Of similar importance is the requirement for a mechanism to sustain the lives of dauers in the presence of an unlimited energy source. A mechanism that is compatible with various experimental conditions is the accumulation of toxic compound(s) resulting from fat metabolism. Thus, in a fashion closely resembling the so‐called “lipotoxicity” in mammalian metabolic syndrome, the enhanced lipogenesis in dauers could lead to deleterious ectopic lipid deposits and a harmful increase in the concentrations of diacylglycerols, ceramides, and other compounds that could interfere with the normal cellular functions (DeFronzo, 2010; Schooneman, Vaz, Houten, & Soeters, 2013). In its current state, our mathematical model strongly supports the possibility of such a mechanism. Furthermore, a number of genes that participate in the longevity assurance in *C. elegans* mutants with reduced insulin signaling seem to act in the detoxification of lipophilic toxic compounds (Gems & McElwee, 2005). This principle may be conserved in the metabolic adaptation to ethanol in dauer larvae. Future studies should refine the model and identify other possible determinants of survival, such as detoxification and mitochondrial recovery.

Taken together, our study provides insights into how dauer larvae utilize environmental sources of energy while maintaining a homeostatic balance through fine regulation of the metabolic mode. This could represent an essential survival strategy and stress resistance mechanism employed by worms and other animals. Furthermore, it may open new avenues for research into how organisms cope with a surplus of energy to prevent the development of metabolic syndrome in higher organisms, including humans.

## 4 EXPERIMENTAL PROCEDURES

A detailed description of all experimental procedures can be found in the Appendix S1.

## CONFLICT OF INTEREST

The authors declare no conflict of interest.

## AUTHOR CONTRIBUTIONS

D.K., S.P., and T.V.K. designed the experiments. D.K. and S.P. conducted phenotypical, fluorescent microscopy, and biochemical experiments. V.G. analyzed desiccation tolerance. R.G. and E.K. contributed CARS microscopy. B.K.R. and A.S. provided MS Western mass spectrometry data. X.Z. and V.Z. performed mathematical modeling. J.L.S. contributed HPLC‐MS analysis. R.H. analyzed microscopy images of mitochondria. All authors discussed the results. D.K., S.P., and T.V.K. wrote the manuscript.

## Supporting information

Appendix S1

## Data Availability

The data that support the findings of this study are available from the corresponding author upon reasonable request.
