# Supplementary material for: Exogenous ethanol induces a metabolic switch that prolongs the survival of Caenorhabditis elegans dauer larva and enhances its resistance to desiccation
Source: Aging Cell. 2020 Sep 8;19(10):e13214. doi: 10.1111/acel.13214 (PMC7576309; doi:10.1111/acel.13214)

Supporting information

Exogenous ethanol induces a metabolic switch that prolongs the survival of *C. elegans* dauer larva and enhances its resistance to desiccation

Damla Kaptan, Sider Penkov, Xingyu Zhang, Vamshidhar R. Gade, Bharath Kumar Raghuraman, Roberta Galli, Júlio L. Sampaio, Robert Haase, Edmund Koch, Andrej Shevchenko, Vasily Zaburdaev and Teymuras V. Kurzchalia

Inventory of the supporting information

1. Experimental procedures
2. References to supporting information
3. Legends to supplementary figures
4. Supplementary figures

1. Experimental procedures

Worm strains and cultivation

Wild-type strain used was *C. elegans* variant Bristol, strain N2. The following single mutant strains were used in this study: DA2579 *sodh-1(ok2799) V*, CB1370 *daf-2(e1370) III*, CB1372 *daf-7(e1372) III*, TJ1052 *age-1(hx546) II*. All *C. elegans* strains were obtained from the Caenorhabditis Genetics Center (CGC). *daf-2(e1370);aak-2(gt33)* double mutant was generated as described previously (Penkov et al., 2018). During this study, the following strains have been generated:

daf-2;aak-2;mitoGFP: Males of *daf-2(e1370); zcIs14 [myo-3::GFP(mit)],* (dubbed *daf-2;mitoGFP*) were crossed to hermaphrodites of *daf-2(e1370);aak-2(gt33)*. Males from the progeny were crossed to the mother strain *daf-2(e1370);aak-2(gt33)*. Eggs from the resulting hermaphrodites were grown at 25˚C and developed into dauers. The dauers were left to recover at 15˚C and eGFP-positive worms were selected and singled. The progeny of these worms was selected based on the fluorescent signal. The presence of *aak-2(gt33)* was tested by PCR.

sodh-1;mitoGFP: Males of *zcIs14 [myo-3::GFP(mit)]* (dubbed *mitoGFP*) were crossed to hermaphrodites of *sodh-1(ok2799)*. Resulting hermaphrodites produced eggs that were singled and selected for *sodh-1(ok2799)* via PCR and *mitoGFP* via fluorescent signal.

Worms were routinely cultured on Nematode Growth Medium (NGM) plates seeded with *E. Coli* NA22 strain(Brenner, 1974). Worms were placed on the plates either as mixed stage populations or as embryos obtained by hypochlorite treatment. Dauers were obtained by 1% SDS treatment of mixed stage populations from

overcrowded plates. Worms were collected from the feeding NGM-agar plates, washed three times with ddH_2O, and resuspended in 10 ml of 1% SDS (w/v) in ddH_2O in 15 ml polypropylene centrifuge tubes (Corning, NY, USA). After 30 minutes of incubation within the SDS solution at 25˚C with shaking, the worms were washed another three times with ddH_2O and placed on agarose plates where the alive worms (dauers) were separated from the dead worms (non-dauers) based on their motility. The temperature-sensitive Daf-c mutants were grown at 15 °C to grow in the reproductive mode and at 25 °C to arrest as dauer larvae.

Worms were cultured on Nematode Growth Medium (NGM) plates seeded with *E. Coli* NA22 strain (Brenner, 1974). Worms were placed on the plates either as mixed stage populations or as embryos obtained by hypochlorite treatment. Dauers were obtained by 1% SDS treatment of mixed stage populations from overcrowded plates. Worms were collected from the feeding NGM-agar plates, washed three times with ddH_2O and resuspended in 10 ml of 1% SDS (w/v) in ddH_2O in 15 ml polypropylene centrifuge tubes (Corning, NY, USA). After 30 minutes of incubation within the SDS solution at 25˚C with shaking, the worms were washed another three times with ddH_2O and placed on agarose plates where the alive worms (dauers) were separated from the dead worms (non-dauers) based on their motility. The temperature-sensitive Daf-c mutants were grown at 15 °C to grow in the reproductive mode and at 25 °C to arrest as dauer larvae.

Dauer survival assay

All dauer survival assays were performed in liquid. The inherent dispersive behavior of dauer larvae obstructed the scoring of survival rates on solid medium as also

described by other groups (Narbonne & Roy, 2006). Dauers were incubated in 10 ml Complete-S medium cultures supplied with designated ethanol concentrations or without ethanol. No bacteria were added. Of note, the Complete-S medium contains phosphates, sulfates, and a mix of trace metals (Stiernagle, 2006). Wherever mentioned, a mixture of the following amino acids and ammonium chloride was provided: L-Alanine, L-Arginine, L-Aspartate, L-Cysteine, L-Glutamate, Glycine, L-Histidine, L-Isoleucine, L-Leucine, L-Lysine, L-Methionine, L-Phenylalanine, L-Proline, L-serine, L-Threonine, L-Tyrosine, L-Valine. All amino acids and the ammonium chloride were in 0.25 µM final concentration, except L-cysteine, which was 0.125 µM. Similarly, wherever mentioned, a mixture of the following vitamins was provided in final concentration: 4-Aminobenzoic acid (0.15 µg/ml), D-Biotin (0.075 µg/ml), Folic acid (0.15 µg/ml), Niacinamide (0.15 µg/ml), D-Pantothenic acid (0.15 µg/ml), Pyridoxal (0.075 µg/ml), Pyridoxine (0.15 µg/ml), Riboflavin (0.15 µg/ml), Thiamine (0.15 µg/ml), D/L-6,8-Thioctic acid (0.075 µg/ml). Cultures were maintained shaking at 25 °C. Every week, aliquots containing around 50 worms were scored for survival. The medium was renewed every week, thus maintaining the concentration of ethanol, amino acids, and vitamins relatively constant and eliminating waste products. Cultures were aerated approximately every 3.5 days.

Desiccation tolerance assay

Dauer larvae were prepared and treated with ethanol as described above. Samples for desiccation were prepared as described before (Erkut et al., 2011). Shortly, dauer larvae were collected in ddH_2O and transferred onto 3.5 cm plastic Petri dishes. The dishes were placed in a chamber with controlled relative humidity (RH) at 98% RH and

incubated for four days. The samples were then either kept at 98% RH or transferred into another chamber at 60% RH for one additional day. Ultimately, the worms were rehydrated with ddH_2O for at least 2 h, transferred to NGM agar plates seeded with *E. coli* NA22 and kept at 15 °C overnight to recover, after which survival was scored.

2-Dimensional difference gel electrophoresis (2D-DIGE)

Fifty micrograms of each protein sample (as determined using RC/DC kit) in urea lysis buffer were labeled with 250 pmol CyDye DIGE Fluor dyes (GE Healthcare, Germany). After labeling, excess dyes were quenched with 10 nmol L-lysine and the samples were reduced in rehydration buffer (7 M urea, 2 M thiourea, 4% CHAPS (w/v), and 50 mM DTT). The samples were pooled and supplemented with ampholytes (BioLytes pH 3–10, BioRad, Germany) in a total volume of 350 µl. This labeled protein mixture was loaded into an immobilized pH gradient strip (linear pH of 3–10) via passive rehydration for 24 h. Following rehydration, isoelectric focusing was performed in a Protean IEF cell (BioRad, Germany) for 55–60 kVh in total. The strip was then equilibrated in equilibration buffer (6 M urea, 2% SDS (w/v), 50 mM Tris, 20% glycerol (v/v), and 130 mM DTT) for 10 min before being placed on a 20 cm wide 12% SDS-polyacrylamide gel. Proteins in the strip, along with PageRuler Plus prestained protein weight marker (Fermentas, USA), were separated by SDS-PAGE at 200 V for 5 h. Finally, the gel was scanned using a Typhoon 9500 Fluo and Phospho Imager (GE Healthcare, Germany) at 100 µm/pixel resolution for Cy2 (488 nm excitation, BP 520/40 emission filter), Cy3 (532 nm excitation, BP 580/30 emission filter), and Cy5 (633 nm excitation, BP 670/30 emission filter) at empirically determined photomultiplier tube voltages. After laser scanning, gels were stained with

Coomassie blue and the spots of interest were cut out. The proteins in these gel slices were extracted and characterized with geLC-MS/MS (Vasilj, Gentzel, Ueberham, Gebhardt, & Shevchenko, 2012).

MS Western quantification of the metabolic enzymes

All reagents were of the analytical grade. LC-MS grade solvents were purchased from Fisher Scientific (Waltham, MA); formic acid (FA) from Merck (Darmstadt, Germany), Complete Ultra Protease Inhibitors from Roche (Mannheim, Germany); Trypsin Gold, mass spectrometry grade, from Promega (Madison). Other common chemicals and buffers were from Sigma-Aldrich (Munich, Germany). Protein quantification was performed using Pierce BCA protein assay kit from Thermo Scientific (Rockford, USA). Ampoules of Pierce BSA standard and isotopically labeled $^{13}C_6{}^{15}N_4$-L-arginine and $^{13}C_6$-L-lysine were purchased from Thermo Scientific (Rockford, USA) and Silantes (Munich, Germany) respectively. Worms were washed twice with M9 buffer, counted, collected and snap-frozen in liquid nitrogen for later analysis. The frozen worms were thawed on ice and crushed using a micro hand mixer (Carl Roth, Germany). The crude extract was centrifuged for 15 min. at 13,000 rpm at 4°C to remove any tissue debris. The clear supernatant was transferred to a fresh Protein Lo-Bind tube (Eppendorf, Hamburg, Germany). The total protein content of the samples was estimated using BCA assay and 15 µg of total protein content was loaded to a precast 4 to 20% gradient 1-mm thick polyacrylamide mini-gels from Anamed Elektrophorese (Rodau, Germany). Separate gels were run for the BSA and isotopically labeled chimeric protein standard. Undetectable proteins or proteins without detectable unique peptides (e.g., GPD-1, GPD-3, HXK-1, SODH-2 SUCL-1,

and SDHD-1) were not included. From the aldehyde dehydrogenase family, only the peptides from ALH-1, ALH-9 and ALH-12 were included for absolute quantification as the contribution of other family members to the total pool of ALHs was very small (Fig. S3). The gels were processed according to the protocol described in (Kumar et al., 2018). Peptide matching was carried out using Mascot v.2.2.04 software (Matrix Science, London, UK) against *Caenorhabditis elegans* (November 2016) proteome downloaded from Uniprot. A precursor mass tolerance of 5 ppm and fragment mass tolerance of 0.03 Da was applied, fixed modification: carbamidomethyl (C); variable modifications: acetyl (protein N terminus), oxidation (M); labels: 13C(6) (K) and 13C(6)15N(4) (R); cleavage specificity: trypsin, with up to 2 missed cleavages allowed. Peptides having the ions score above 15 were accepted (significance threshold $p < 0.05$). The chromatographic alignment and feature detection were carried using Progenesis LC-MS v.4.1 (Nonlinear Dynamics, UK). The absolute quantification was performed using in-house software. The label-free quantification and subsequent statistical analysis were performed using MaxQuant (v 1.6.0.16) (Cox & Mann, 2008) and Perseus (v1.6.2.1) (Tyanova et al., 2016), respectively.

^{14}C-Ethanol Labeling

Ethanol labeling was performed by directly incubating wild-type, *sodh-1(ok2977)*, and *age-1(hx546)* dauers with 10 μCi of [1-^{14}C-EtOH] (Biotrend, Germany), in 10 ml complete S-medium. After the incubation was completed, worms were washed three times with ddH_2O and homogenized by five rounds of freezing in liquid nitrogen and thawing in an ultrasound water bath. Organic compounds were extracted from homogenized samples according to a standard method (Bligh & Dyer, 1957). Aqueous

fractions were dissolved in 50% CH_3OH, while organic fractions were dissolved in $CHCl_3$:CH_3OH (1:2, v/v). Total radioactivity in each sample was measured using a scintillation counter. Samples were normalized according to the number of worms, and loaded on glass HPTLC plates (Merck, Darmstadt, Germany) covered with silicate. 2D-TLC of aqueous fractions for the visualization of hydrophilic metabolites was done using 1-propanol–methanol–ammonia (32%)–water (28:8:7:7, v/v/v/v) as 1st system and 1-butanol–acetone–glacial acetic acid–water (35:35:7:23, v/v/v/v) as the 2nd. Lipids in organic fractions were analyzed by one-dimensional thin layer chromatography (1D-TLC) with the solvent system chloroform-methanol-water (45:18:3, v/v/v). TLC plates containing radioactive samples were sprayed with EN^{3}HANCE spray surface autoradiography enhancer (Perkin Elmer, Waltham, MA, USA) and exposed to X-ray film (Kodak Biomax MR, Sigma-Aldrich, Taufkirchen, Germany).

HPLC-MS analysis of *C. elegans* metabolites

About 20,000 dauer larvae were pelleted, washed three times, re-suspended in 500 µl of LC-MS-grade H_2O (Merk, Darmstadt, Germany), and snap-frozen in liquid nitrogen. The frozen pellet was freeze-sonicated 3-4 times until the lysate became homogeneous. A volume of lysate corresponding to 50 µg total protein content was diluted with LC-MS-grade H_2O to a total volume of 200 µl. 750 µl of chloroform-methanol (1:2, v/v) were added and the mixture was shaken for 20 min. 250 µl of $CHCl_3$ were added followed by vigorous shaking. Finally, 250 µl of LC-MS-grade H_2O were added followed by vigorous shaking and centrifugation at 16,000 RCF for 5 min. for phase separation. The upper phase (aqueous phase) was collected and dried under

nitrogen flow. The extract was re-suspended in 120 µl of LC-MS-grade H_2O. For HPLC-MS analysis, 20 µl of the suspension were mixed to 80 µl of 95% acetonitrile and 20 µl of this mixture were injected into a high performance liquid chromatography (HPLC) system (1200 Agilent) coupled online to G2-S QTof (Waters). For chromatographic separation a Bridge Amide 3.5ul (2.1x100mm) column (Waters) was used. The mobile phase was composed of 95% acetonitrile, 0.5 mM CH_3COONH_4, 0,056 % NH_4OH (eluent A), and 40% acetonitrile, 0.5 mM CH_3COONH_4, 0,056 % NH_4OH (eluent B). The following gradient program was used: Eluent B: from 0% to 100% for 15 min; 100% from 15min to 18min; 0% from 18 min to 25 min. The flow rate was set at 0.5 ml/min. The spray voltage was set at 2.5 kV and the source temperature at 150°C. Nitrogen was used as both cone gas (50 L/h) and desolvation gas (1000 L/h). The desolvation temperature was 500°C. Spectra was acquired in negative ionization polarity. The peak areas of the different metabolites were extracted from the chromatogram using MassLynx software (Waters).

CARS microscopy

The imaging of lipid droplets was performed by coherent anti-Stokes Raman scattering (CARS) microscopy (Camp Jr & Cicerone, 2015; Jungst, Winterhalder, & Zumbusch, 2011). CARS was detected using a multiphoton scanning microscope coupled with two near-infrared picosecond fiber lasers. The optical microscope was an upright Axio Examiner Z.1 equipped with a laser scanning module LSM 7 (all from Carl Zeiss Microscopy GmbH, Jena, Germany) and multiple detectors in the non-descanned configuration. The laser source used to excite the CARS signal (Femto Fiber pro TNIR from Toptica Photonics AG) is tunable in the range 850 - 1100 nm and has a pulse

length of 0.8 ps. The wavelength was set to 1005 nm (emitted power at the source:1.5 mW), to resonantly excite the symmetric stretching vibration of methylene groups at 2850 cm^{-1}. The CARS signal was collected in transmission mode and selected using a BP filter (640 ± 7) nm. A water immersion objective W Plan-Apochromat 20×/1.0 (Carl Zeiss Microscopy GmbH) was used. Due to the transmission of optical elements, the laser power in the sample was 52 mW. An automatic tiling procedure enabled by the microscope software ZEN was used for the acquisition of images larger than the field of view of the microscope objective. Multiple optical planes were acquired in z-stacks covering all worm tissues containing lipid droplets. The total area of the lipid droplets detected by CARS was calculated for all optical planes using Fiji (Schindelin et al., 2012).

Imaging of mitochondria

For the visualization of mitoGFP by confocal microscopy, worms were mounted on 2% agarose pads on glass slides (Thermo scientific, Superfrost Plus) and anesthetized with 500 mM levamisole in complete-S medium. The liquid was aspirated and the pads were covered with coverslips (with 0.17 +/- 0.005 mm cover slips (Menzel-Glaeser). The mitoGFP was visualized with a Zeiss LSM 880 scanning confocal microscope equipped with a Zeiss i LCI Plan-Neofluar 63x 1.3 Imm Korr DIC objective. eGFP was excited at 488 nm, and fluorescence was detected at the emission band of 490-540 nm. Depending on the depth of the tissue occupied by eGFP-marked mitochondria, single or several adjacent optical planes were acquired. The mean circularity of mitochondria was determined using shape descriptor analysis in Fiji (Schindelin et al., 2012) over all objects in one image (for single planes) or a maximum z projection (for multiple

planes). The circularity of a particle was defined as $4\pi \times [Area] / [Perimeter]^2$, with a value of 1.0 indicating a perfect circle. The nuclei, which are also marked with eGFP, were manually annotated and removed from the analysis in advance.

This analysis was implemented as a macro for the current version of Fiji 2.0.0-rc-69/1.52p. The code of the macro is available under the following link: https://doi.org/10.5281/zenodo.3918953.

Mathematical modeling

To model the metabolic pathway (see Fig. 5A) of dauer larvae, we used the standard way of describing chemical reactions as a system of ordinary differential equations for the concentrations of participating components (Murray, 1989). For most of the reactions, we applied standard equations of chemical kinetics with forward and backward rates:

$$\frac{da}{dt} = -(k_1 + k_4)a + k_2 l + j_{in} \quad (1)$$

$$\frac{dl}{dt} = k_1 a - k_2 l \quad (2)$$

$$\frac{dc}{dt} = k_3 k_2 l \quad (3)$$

$$\frac{dm}{dt} = -k_{d1}\Theta(k_4 a - j_{min})m - k_{d2}\Theta(c_h - c)m \quad (4)$$

$$\Theta = \begin{cases} 1 & if\ x \geq 0 \\ 0 & if\ x \leq 0 \end{cases} \quad (5)$$

Here *a* and *l* represented the concentrations of the acetate and lipid respectively, where k_1 and k_2 are the forward and backward reaction rates for acetate to lipid conversion (see below). Toxic compound concentration was denoted by *c* and it is produced from the lipolysis with the rate k_3. The consumption of acetate was also unidirectional with

the rate k_4 (see below). If there was exogenous ethanol, its presence was included via a constant influx j_{in} of acetate. The wellbeing of mitochondria was represented by a number denoted by m, which took values from 1 (completely functional mitochondria) to 0 (mitochondria completely damaged). Mitochondria could be damaged with a rate k_{d1} if the carbohydrate production, $k_4 a$, fell below a threshold j_{min}, or with a rate k_{d2} when the toxic compound c accumulated above a certain threshold c_h. Θ in Eq.(4) denotes the Heaviside step function as defined in Eq.(5).

While most of the reaction rates for simplicity were taken as constants, there were some rates that depended on chemical variables. One example was the dependence of k_4 on m, where we assumed that energy production requires functioning mitochondria and thus used a simple linear relation:

$$k_4 = \tilde{k}_4 m,$$

where $\tilde{k}_4$ was a constant. Another rate that depended on concentrations was k_1 denoting the conversion rate from acetate to lipids. It reflected the fact that there was a certain maximum amount of lipid l_s that could be possibly stored in a single worm and thus:

$$k_1 = \tilde{k}_1 \frac{l_s - l}{l_1 + (l_s - l)}$$

Here l_1 was a constant determining at which lipid concentrations the conversion started to slow down and $\tilde{k}_1$ was also a constant. Finally, we chose k_2 rate to be of the Michaelis-Menten type:

$$k_2 = \tilde{k}_2 \frac{1}{l_2 + l}$$

where $\tilde{k}_2$ and l_2 were constants.

Overall the above system of equations contains six independent parameters and requires the knowledge of the initial conditions for all four participating components. While measuring most of the involved rates and concentrations is potentially possible, it certainly goes beyond the scope of this manuscript. Instead, we chose the following strategy: We assumed that the model could predict a certain lifespan of wild-type or *daf-2* dauer (for simplicity, we do not mathematically distinguish between wild-type and *daf-2* because they have similar lifespans). We wanted to check, if there existed a region in the parameter space of the model, where, by having all parameters fixed and only changing those that would correspond to a certain mutation or a presence or absence of ethanol, we could reproduce the changes in the lifespan with respect to the wild-type/*daf-2* as was seen in the experiments.

Parameters used:

$\tilde{k}_{1(daf2)}/\tilde{k}_4$	9.0	l_1/l_2	0.1
$\tilde{k}_{1(age1)}/\tilde{k}_4$	3.0	j_{min}/l_2k_4	0.05
$\tilde{k}_{2(daf2)}l_2/\tilde{k}_4$	0.6	c_h/l_2	0.1
$\tilde{k}_{2(aak2)}l_2/\tilde{k}_4$	6.0	$a(t=0)/l_2$	0.2
$\tilde{k}_3/\tilde{k}_4$	0.01	$l(t=0)/l_2$	6.0
l_s/l_2	8.0	$j_{in}/l_2\tilde{k}_4$	0/0.09

To model the pathway in *daf-2;aak-2* and *age-1* mutants, we modified the rates of the reactions regulated by AAK-2 and AGE-1 as illustrated on Fig. 5A. In the case of *daf-2;aak-2*, the only part in the model that is modified by the *aak-2* mutation is an increased $\tilde{k}_2$. Correspondingly, we chose $\tilde{k}_2$ to be ten times larger than for a wild-

type/*daf-2* dauer. Regarding *age-1* mutants, we exploited the possibility that they may have a reduced acetate to lipid transformation rate, which reduces both lipid accumulation and toxic production. We tested this assumption by choosing k_1 of age-1 mutant to be one-third of that in the wild-type/*daf-2* dauer. To solve the system of differential equations, we used standard numerical integration tools in MATLAB.

2. References to supporting information

Bligh, E. G., & Dyer, W. J. (1957). A rapid method of total lipid extraction and purification. *Can J Biochem Physiol, 37*, 911-917.

Brenner, S. (1974). The genetics of Caenorhabditis elegans. *Genetics, 77*(1), 71-94.

Camp Jr, C. H., & Cicerone, M. T. (2015). Chemically sensitive bioimaging with coherent Raman scattering. *Nature Photonics, 9*(5), 295-305.

Cox, J., & Mann, M. (2008). MaxQuant enables high peptide identification rates, individualized p.p.b.-range mass accuracies and proteome-wide protein quantification. *Nat Biotechnol, 26*(12), 1367-1372. doi:10.1038/nbt.1511

Erkut, C., Penkov, S., Khesbak, H., Vorkel, D., Verbavatz, J. M., Fahmy, K., & Kurzchalia, T. V. (2011). Trehalose renders the dauer larva of Caenorhabditis elegans resistant to extreme desiccation. *Current biology : CB, 21*(15), 1331-1336. doi:10.1016/j.cub.2011.06.064

Jungst, C., Winterhalder, M. J., & Zumbusch, A. (2011). Fast and long term lipid droplet tracking with CARS microscopy. *J Biophotonics, 4*(6), 435-441. doi:10.1002/jbio.201000120

Kumar, M., Joseph, S. R., Augsburg, M., Bogdanova, A., Drechsel, D., Vastenhouw, N. L., . . . Shevchenko, A. (2018). MS Western, a Method of Multiplexed Absolute Protein Quantification is a Practical Alternative to Western Blotting. *Molecular & cellular proteomics : MCP, 17*(2), 384-396. doi:10.1074/mcp.O117.067082

Murray, J. D. (1989). Mathematical biology, vol. 19 of Biomathematics: Springer, Berlin, Germany.

Narbonne, P., & Roy, R. (2006). Inhibition of germline proliferation during C. elegans dauer development requires PTEN, LKB1 and AMPK signalling. *Development, 133*(4), 611-619. doi:10.1242/dev.02232

Penkov, S., Erkut, C., Oertel, J., Galli, R., Vorkel, D., Verbavatz, J.-M., . . . Kurzchalia, T. V. (2018). An insulin, AMPK, and steroid hormone-mediated metabolic switch regulates the transition between growth and diapause in C. elegans. *bioRxiv*. doi:10.1101/323956

Schindelin, J., Arganda-Carreras, I., Frise, E., Kaynig, V., Longair, M., Pietzsch, T., . . . Cardona, A. (2012). Fiji: an open-source platform for biological-image analysis. *Nature methods, 9*(7), 676-682. doi:10.1038/nmeth.2019

Stiernagle, T. (2006). Maintenance of C. elegans. *WormBook : the online review of C. elegans biology*, 1-11. doi:10.1895/wormbook.1.101.1

Tyanova, S., Temu, T., Sinitcyn, P., Carlson, A., Hein, M. Y., Geiger, T., . . . Cox, J. (2016). The Perseus computational platform for comprehensive analysis of (prote)omics data. *Nature methods, 13*(9), 731-740. doi:10.1038/nmeth.3901

Vasilj, A., Gentzel, M., Ueberham, E., Gebhardt, R., & Shevchenko, A. (2012). Tissue proteomics by one-dimensional gel electrophoresis combined with label-free protein quantification. *J Proteome Res, 11*(7), 3680-3689. doi:10.1021/pr300147z

Legends to supplementary figures

Figure S1. Translational activation during exit from dauer state and induction of alcohol-metabolizing enzymes by ethanol.

2D-DIGE of proteins derived from wild-type dauers treated or untreated with food on solid medium. Worms exit from dauer state and this process is accompanied by a global translational activation as evidenced by the increase in multiple proteins (in green) in food-treated worms. Representative images of two experiments.

Figure S2. Treatment with ethanol leads to the accumulation of trehalose and a higher turnover of amino acids and nucleotides.

HPLC-MS analysis of the levels of various metabolites in untreated and ethanol-treated wild-type dauers over time. Two biological replicates per time point are displayed.

Figure S3. ALH-1 is the major aldehyde dehydrogenase that is upregulated upon ethanol treatment.

Relative proteomic quantification of the normalized abundances of all detectable ALH members in untreated and ethanol-treated dauer larvae. The peptide abundances were summed and normalized according to the abundance of the spiked-in BSA standard. Error bars, ± SD of two biological and two technical replicates.

Figure S4. Dauers of *daf-2* survive moderately longer than wild-type dauers with or without ethanol.

The plot combines the mean survival rates of wild-type, *daf-2*, *daf-2;aak-2*, and *age-1* dauers treated or untreated with 1 mM ethanol that are displayed in **Fig. 3D** and **F**.

Figure S5. Exogenous ethanol rescues the desiccation tolerance of *daf-2;aak-2*.

Mean survival of *daf-2* and *daf-2;aak-2* dauers untreated or incubated with 1 or 85 mM ethanol followed by desiccation at various relative humidities (RH). Error bars ± SD of two biological with two technical replicates. For *daf-2* at 98%RH, n = 291 (day 0), 678 (day 3, no ethanol), 694 (day 3, 1 mM ethanol), 882 (day 3, 85 mM ethanol). For *daf-2(e1370)* at 60% RH, n = 388 (day 0), n = 833 (day 3, no ethanol), n = 804 (day 3, 1 mM ethanol), n = 686 (day 3, 85 mM ethanol). For *daf-2;aak-2* at 98%RH, n = 504 (day 0), n = 533 (day 3, no ethanol), n = 260 (day 3, 1 mM ethanol), n = 1343 (day 3, 85 mM ethanol). For *daf-2;aak-2* at 60%RH, n = 388 (day 0), n = 282 (day 3, no ethanol), n = 331 (day 3, 1mM ethanol), n = 859 (day 3, 85mM ethanol).

Figure S6. The addition of exogenous amino acids and vitamins does not enhance the survival rate of dauers treated with ethanol.

Survival rates of wild-type dauers treated with ethanol, amino acids, and vitamins. Means ± SD of two-three biological replicates.

Figure S7. Mathematical modeling of survival rates in *daf-2;aak-2* and *age-1* mutants. Co-plotted are several other trends: “Acetate” is the combined entity representing the free acetic acid and the acetyl-CoA produced in the pathway. “Lipid”

represents the bulk complex lipids, mainly TAGs, derived from the fatty acid component. “Toxic” comprises the putative fatty acid-derived toxic compound(s). “Mito” is defined by the degree of activity of the mitochondria and serves as a proxy to the survival rate.

A. Model-predicted survival rates of *daf-2;aak-2* dauers without ethanol.

B. Model-predicted survival rates of *daf-2;aak-2* dauers treated with ethanol.

C. Model-predicted survival rates of *age-1* dauers without ethanol.

D. Model-predicted survival rates of *age-1* dauers treated with ethanol.

Figure S8. *age-1* mutant dauer larvae have diminished incorporation of ^{14}C-ethanol into lipids. Scintillation counting of radioactivity in the lipid-containing organic fraction and aqueous fraction (containing hydrophilic metabolites such as amino acids, sugars, nucleotides, etc.) of extracts from ^{14}C-ethanol labeled wild-type and *age-1* dauer larvae. Data obtained from two experiments, a total of 4 biological replicates, two technical replicates each. Error bars, +SD. ** $p<0.01$; ns - no significant difference determined by Student's *t*-test.

Supplementary Figures

Figure S1

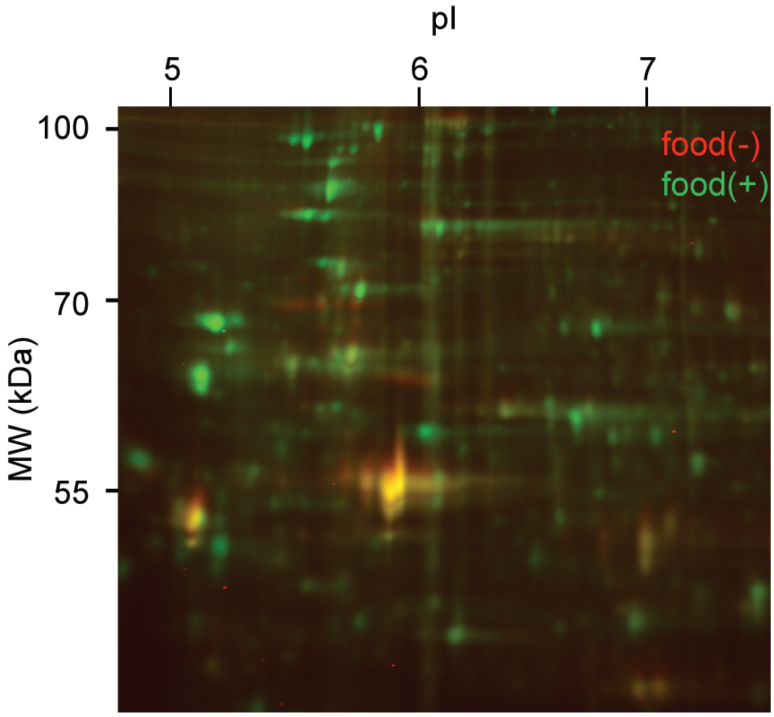

Figure S2

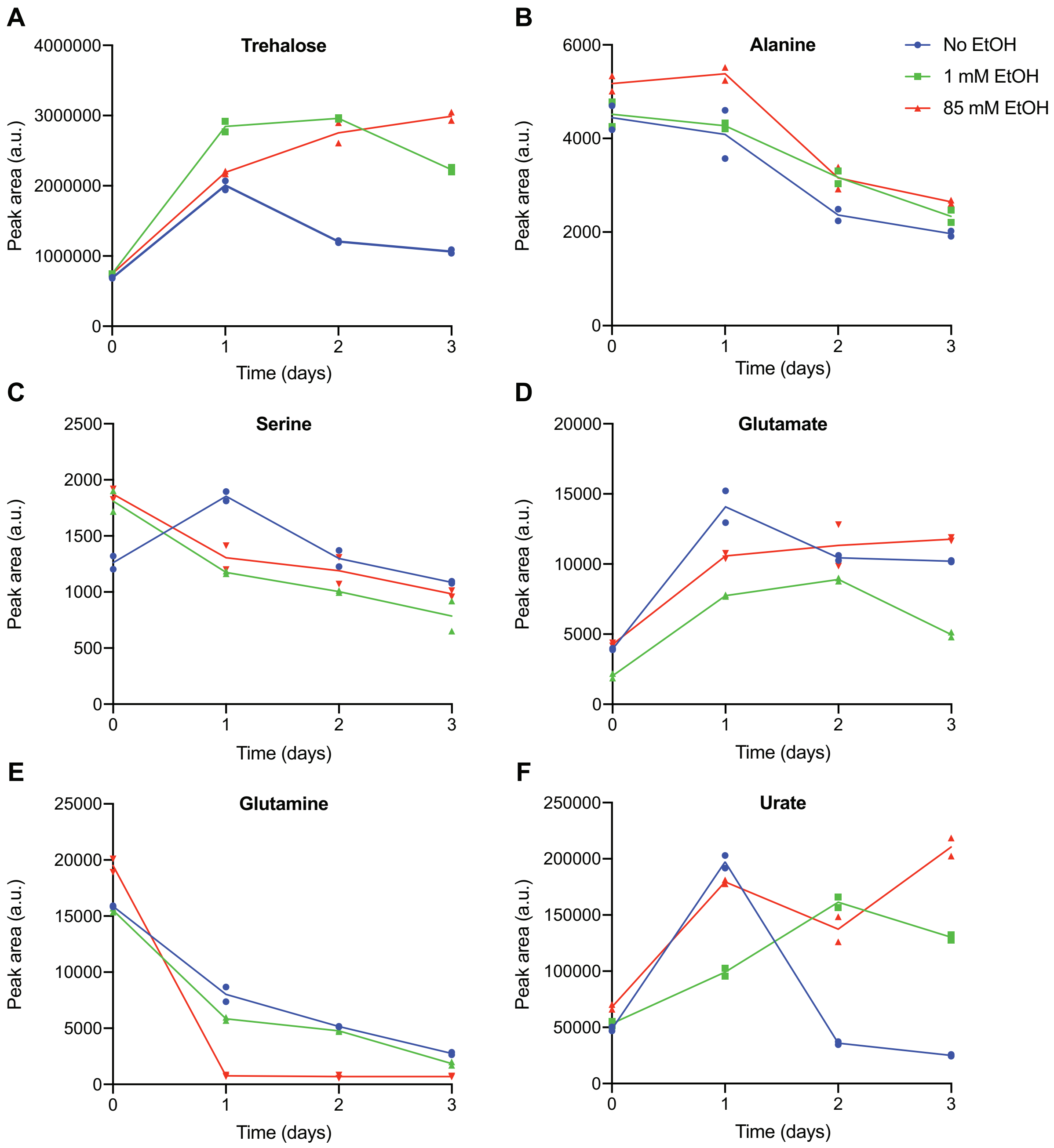

A
Trehalose
Peak area (a.u.)
4000000
3000000
2000000
1000000
0
0
1
2
3
Time (days)
B
Alanine
Peak area (a.u.)
6000
4000
2000
0
Time (days)
No EtOH
1 mM EtOH
85 mM EtOH
C
Serine
2500
2000
1500
1000
500
D
Glutamate
20000
15000
10000
5000
E
Glutamine
25000
20000
15000
10000
5000
F
Urate
250000
200000
150000
100000
50000

Figure S3

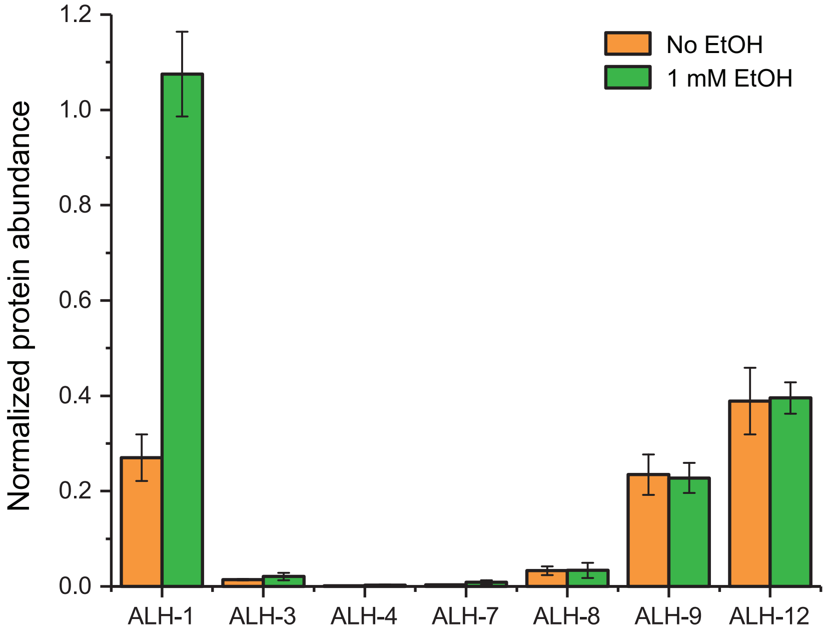

Figure S4

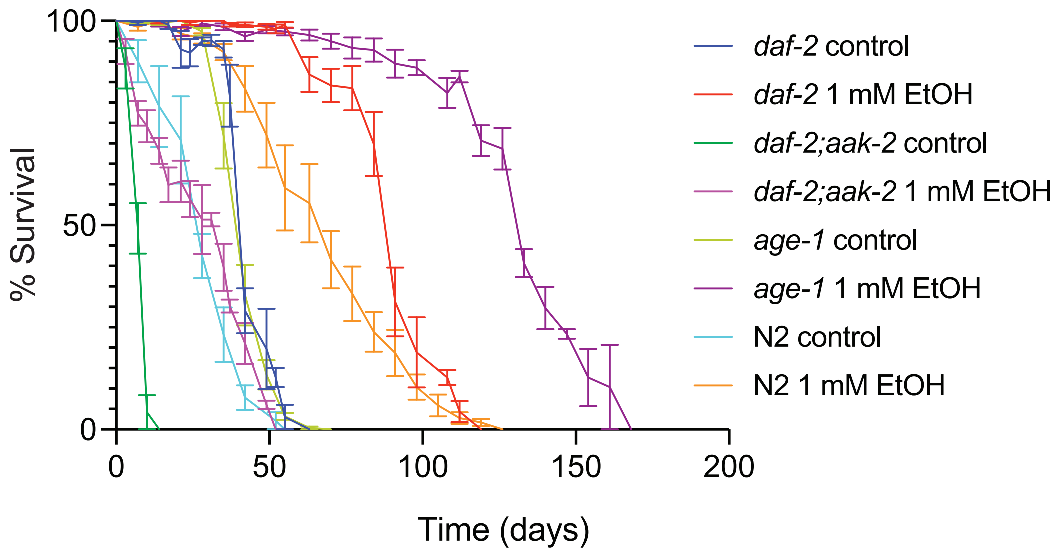

Figure S5

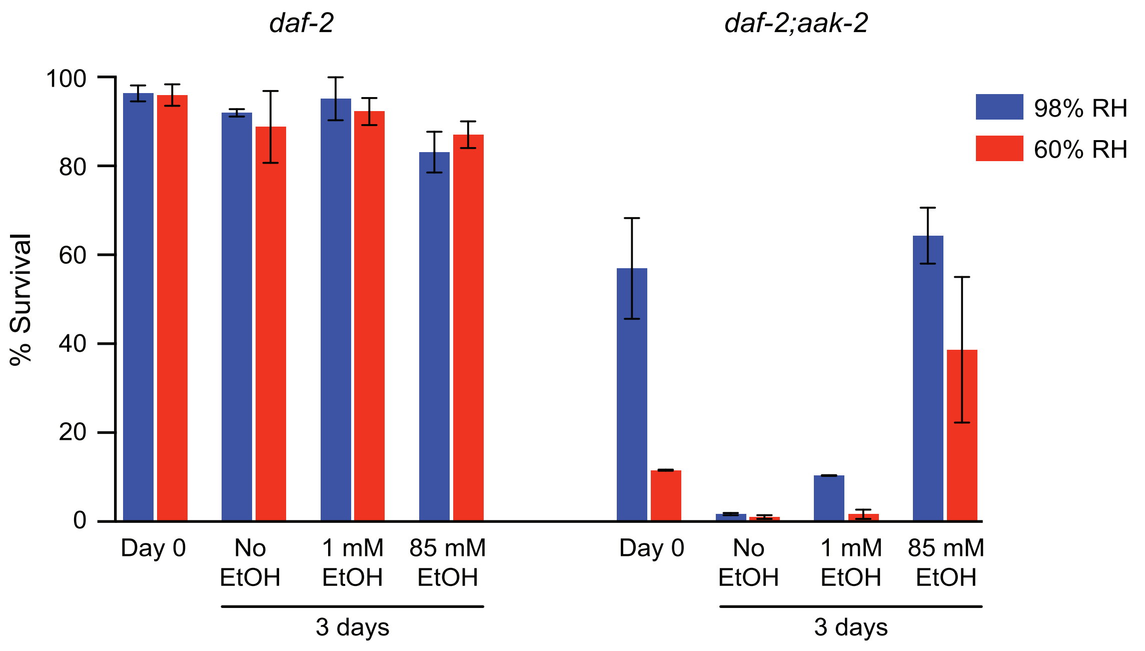

Figure S6

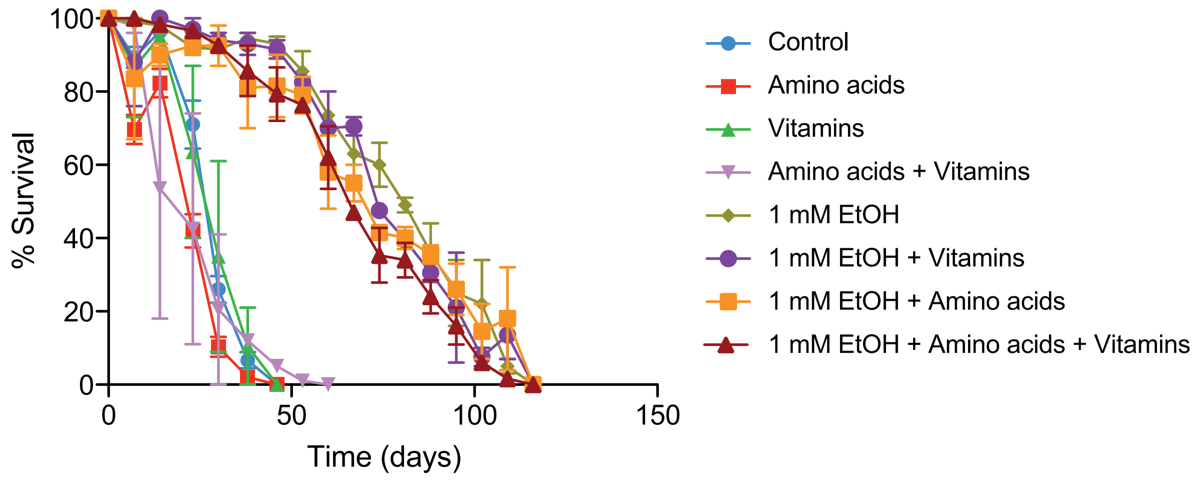

Figure S7

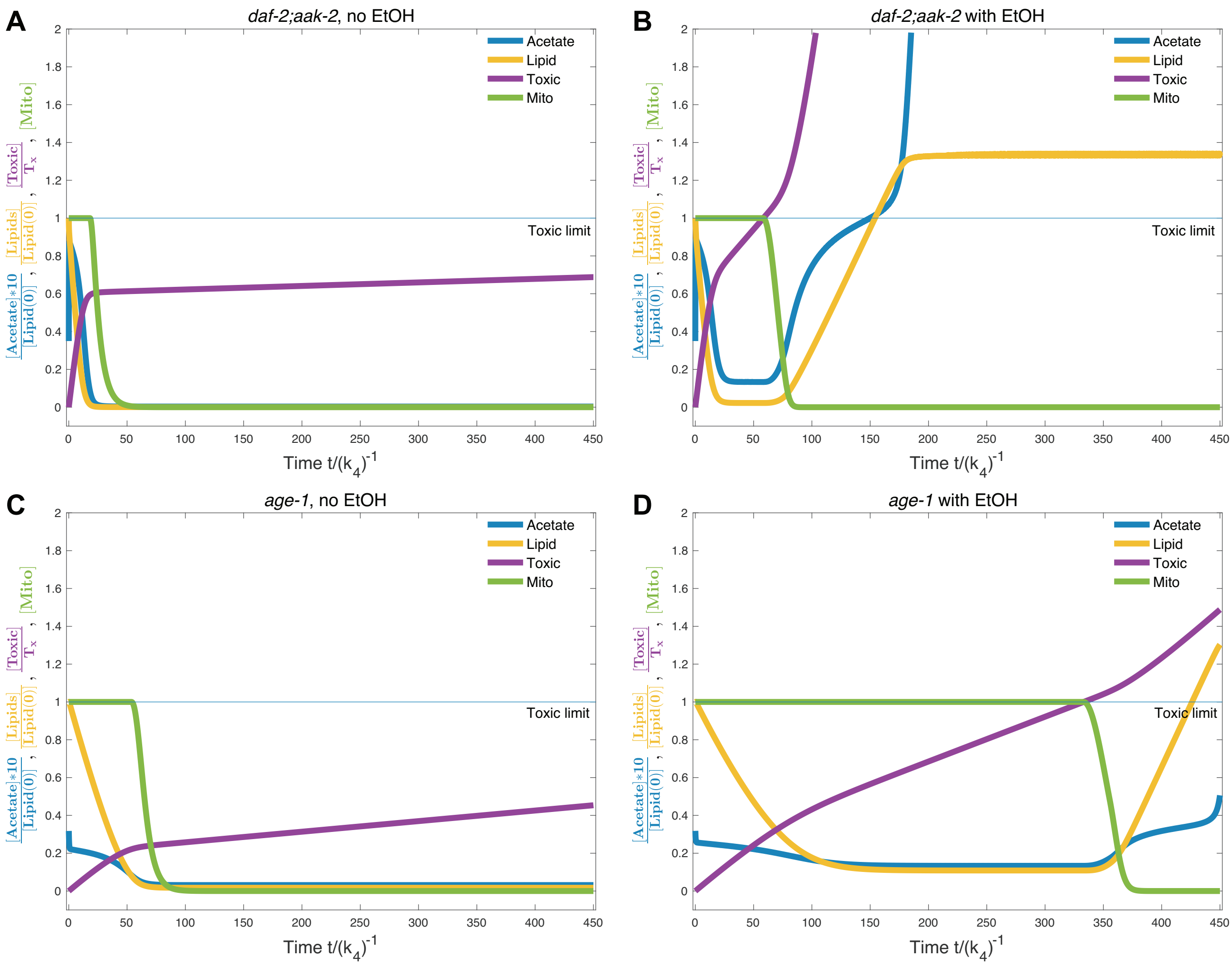

Figure S8

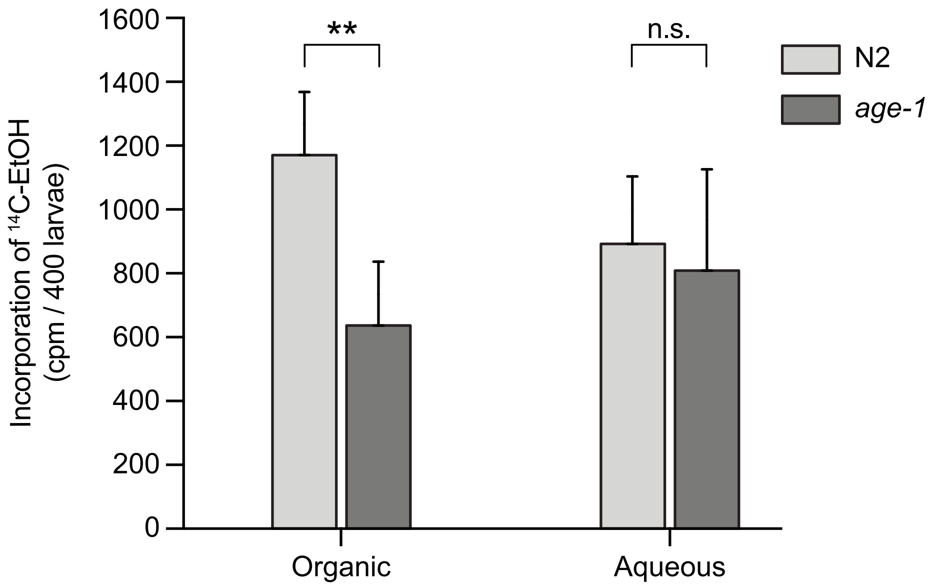

Supplement: Supplementary file 1 — Appendix S1 [file ACEL-19-e13214-s001.pdf]
